# Prolonged production of ^14^C during the ~660 BCE solar proton event from Japanese tree rings

**DOI:** 10.1038/s41598-019-57273-2

**Published:** 2020-01-20

**Authors:** Hirohisa Sakurai, Fuyuki Tokanai, Fusa Miyake, Kazuho Horiuchi, Kimiaki Masuda, Hiroko Miyahara, Motonari Ohyama, Minoru Sakamoto, Takumi Mitsutani, Toru Moriya

**Affiliations:** 1 Faculty of Science, Yamagata University, 1-4-12 Kojirakawa-machi, Yamagata, 990-8560 Japan; 2 Institute for Space-Earth Environmental Research, Nagoya University, Furo-cho, Chikusa-ku, Nagoya, 464-8601 Japan; 3 Graduate School of Science and Technology, Hirosaki University, 3 Bunkyo-cho, Hirosaki, 036-8561 Japan; 4 Humanities and Sciences/Museum Careers, Musashino Art Universally, 1-736 Ogawa-cho, Kodaira, Tokyo, 187-8505 Japan; 5 Botanical Gardens, Tohoku University, 12-2 Kawauchi Aoba-ku, Sendai, 980-0862 Japan; 6 National Museum of Japanese History, 117 Jonai-cho, Sakura, 285-8502 Japan; 7 National Institutes for Cultural Heritage, Nara National Research Institute for Cultural Properties, 2-9-1 Nijo-cho, Nara, 630-8577 Japan; 8 The Graduate University for Advanced Studies, SOKENDAI, 117 Jonai-cho, Sakura, 285-8502 Japan

**Keywords:** Space physics, Solar physics

## Abstract

Annual rings record the intensity of cosmic rays (CRs) that had entered into the Earth’s atmosphere. Several rapid ^14^C increases in the past, such as the 775 CE and 994CE ^14^C spikes, have been reported to originate from extreme solar proton events (SPEs). Another rapid ^14^C increase, also known as the ca. 660 BCE event in German oak tree rings as well as increases of ^10^Be and ^36^Cl in ice cores, was presumed similar to the 775 CE event; however, as the ^14^C increase of approximately 10‰ in 660 BCE had taken a rather longer rise time of 3–4 years as compared to that of the 775 CE event, the occurrence could not be simply associated to an extreme SPE. In this study, to elucidate the rapid increase in ^14^C concentrations in tree rings around 660 BCE, we have precisely measured the ^14^C concentrations of earlywoods and latewoods inside the annual rings of Japanese cedar for the period 669–633 BCE. Based on the feature of ^14^C production rate calculated from the fine measured profile of the ^14^C concentrations, we found that the ^14^C rapid increase occurred within 665–663.5 BCE, and that duration of ^14^C production describing the event is distributed from one month to 41 months. The possibility of occurrence of consecutive SPEs over up to three years is offered.

## Introduction

Since the phenomenon of rapid increase in CR intensity in 775 CE was solved^1–3^, ^14^C analysis in annual rings has played a major role in searching for another rapid ^14^C increase event^4,5^. While such events have been suggested to originate from an extreme SPE^6–10^, the other type with a rather long period of ^14^C increase has recently been reported in German oak tree rings^11^. It is the ^14^C increase in ca. 660 BCE whose ^14^C increment is comparable to that of the 775 CE event. Its rise time of 3 to 4 years is, however, longer than that of approximately 1 year in the 775 CE event.

There are several possible CR sources capable of such extraordinary ^14^C increment, i.e. galactic cosmic rays (GCRs) modulated by a variation in the interplanetary magnetic fields due to solar activities, gamma-rays from supernova (SN) and gamma-ray burst (GRB), and an extreme SPE. Recently, O’Hare *et al*. showed sharp increases of ^10^Be and ^36^Cl concentrations with high-resolution in Greenland ice cores around 2610BP (~660 BC)^12^. We name the phenomenon the “~660 BCE event” including the ^14^C increase at ca. 660 BCE.

O’Hare *et al*. mentioned that the solar modulation of GCRs was not suitable to explain the enhancement of ^10^Be, ^36^Cl, and ^14^C concentrations even by decreased solar activities with showing the significant large excesses against the typical amplitude of an 11-year solar cycle^12^. The increases of ^14^C coincide with those of the ice core-based radionuclides, exceeding the variations of 11-year cycle in the range of 4‰^13^. Also, both SN and GRB were inappropriate for the ~660 BCE event, as the large amount of ^10^Be were evidently detected in the ice cores. It is expected that undetectable amount of ^10^Be atoms are produced by the gamma-ray origin^12,14^. According to energy spectrum estimation based on the measurements of ^10^Be and ^36^Cl in ice core, the origin of the ~660 BCE event was suggested to be an extreme SPE with very hard energy spectrum which is an order of magnitude larger than the largest SPE during the instrumental era^12^.

O’Hare *et al*., also, mentioned that the 2.3-yr long peak of the ^10^Be in the ~660 BCE event is likely to have been caused by a rapid production rate increase and a residence time in the atmosphere due to the subsequent transport from the stratosphere to the deposition^12^. However, it is still unclear whether the events, in particular on the longer rise time of ^14^C, are triggered by a single short pulse of a huge SPE or by a consecutive occurrence of considerably large SPEs, while Güttler *et al*. suggest a short duration less than 1 year for the 775 CE event using a carbon cycle box model^7^. Precise ^14^C profiles of the ~660 BCE event will provide us a clue to figure out the origin of ^14^C increase with a rather long rise time. Hence, we carried out accurate ^14^C measurements with higher time resolution using Japanese tree rings. We present the precise ^14^C time profile, and its production profile calculated quantitatively using a carbon cycle box model.

## Results

**Figure 1:**
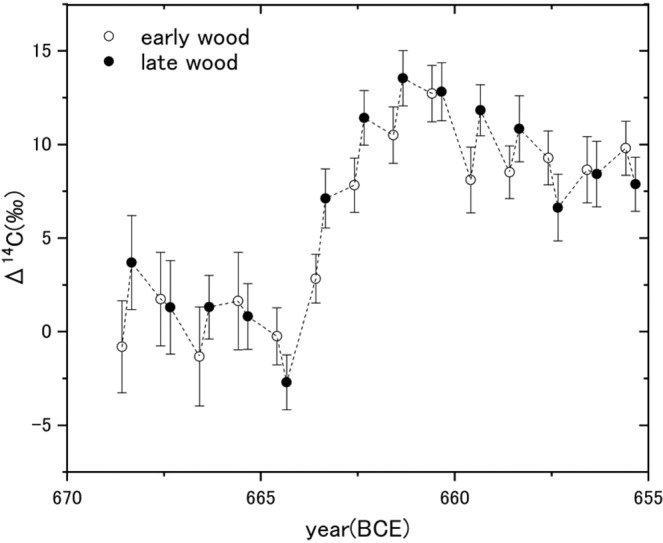
Measured ^14^C concentrations (Δ^14^C) in earlywoods (open circles) and latewoods (solid circles) of Choukai–Jindai cedar annual rings in 669–656 BCE. Since earlywoods and latewoods generally form in spring-to-summer and in summer-to-autumn, respectively, the time resolution of the obtained data set is not strictly half a year. Every earlywoods and latewoods are plotted at 1st June and 1st September, respectively.

We investigated ^14^C concentrations for the earlywoods and latewoods for the period 669–633 BCE in *Cryptomeria japonica* dug out from Choukai volcano in northern Japan (Fig. S1), known locally as “Choukai–Jindai Cedar” (see methods for the dating of the sample and Fig. S2 for ^14^C profile). Figure 1 shows the ^14^C concentrations using the notation of Δ^14^C by Stuiver and Polach^15^ for 669–656 BCE. The Δ^14^C profile shows a rapid increase of 9.8 ± 2.2‰ from the latewood in 665 BCE (plotted in 664.3 BCE in the figure) to the latewood in 664 BCE, going up to a whole of 16.3 ± 2.1‰ by a gradual increase until the latewood in 662 BCE, after which the increment gradually decreased. On the other hand, Δ^14^C in every earlywood of 663–661 BCE were comparable to those in every latewood of the preceding year. This feature is considered to reflect a seasonal variation of ^14^C concentration in the atmosphere, i.e. ^14^C concentrations during the period when latewoods ring formed (August–September) are higher than those of earlywoods (April–July), as stratosphere–troposphere exchange occurs strongly during spring/summer of the Northern Hemisphere^16,17^.

**Figure 2:**
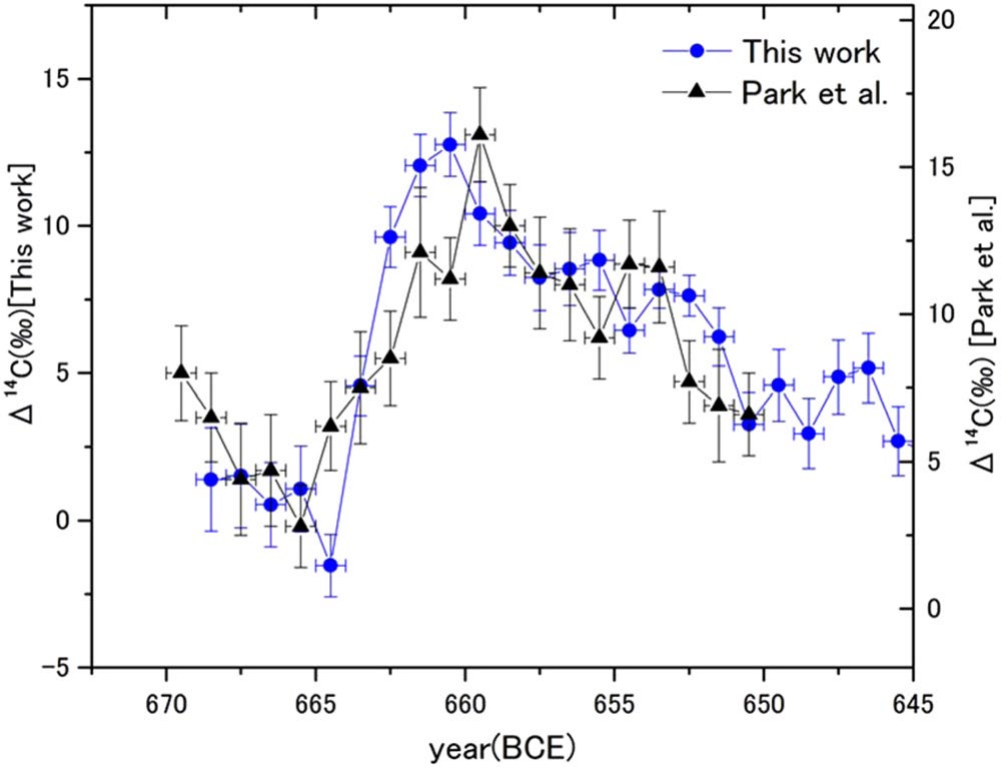
Comparison of annual Δ^14^C profiles between the Choukai–Jindai cedar (blue solid circles) and the German oak (black solid triangles) (Park *et al*.^11^) in 670–646 BCE. Δ^14^C values of the Choukai–Jindai cedar show averages of earlywoods and latewoods. For series comparison of the two series, the vertical axis of the Choukai series is shifted −2‰ from that of the German oak series. The increments from the minimum to the peak in the Choukai–Jindai cedar and German oak series are (14.3 ± 1.5)‰ over 4 years and (13.3 ± 2.1)‰ over 6 years, respectively.

Figure 2 shows a comparison of the annual Δ^14^C data sets for 670–646 BCE between the Choukai–Jindai cedar and the German oak rings previously published^11^. In the comparison, our data were averaged to an annual resolution. Increments from the minimum to the peak of both tree series were mutually comparable. In 669–651 BCE, the average Δ^14^C values of the German oak was (8.9 ± 0.4)‰, which is (1.9 ± 0.5)‰ higher than those of the Choukai–Jindai cedar; the disparity is considered as a kind of regional offset relating to the location of Japanese archipelago on the fringe of the pacific ocean^18^. However, the peak position of Δ^14^C from the German oak seemed to lie one year behind that of the Choukai–Jindai cedar. Although we could not compare the two series directly as the annual data was not a simple average of earlywoods and latewoods, an age mistake of each dendro-model, or a difference of the physiology between oaks and cedars ring growth (see Methods), was taken as a possible reason.

**Figure 3:**
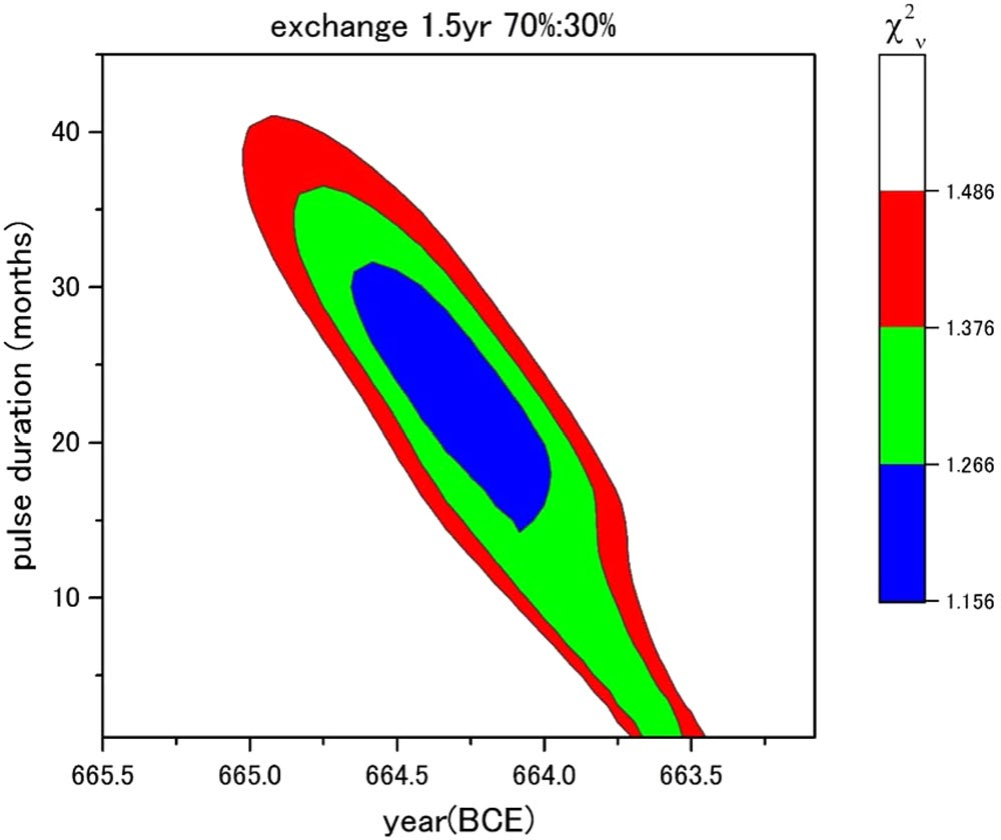
Contour map of reduced χ_ν_^2^ values as a function of pulse duration and pulse start date for the pulse height including the best-fitted single-pulsed event. The conditions of fitting calculation in the 11-box model are 1.5 years, and 70%: 30% for the exchange time and share rate of input ^14^C production between the stratosphere and troposphere, respectively. Outside of the red region is rejected with 95% confidence level.

The Δ^14^C variation appears to be a profile due to a distinct ^14^C input with a duration of longer than a year receiving the global carbon cycle. Employing the 11-box model by Güttler *et al*.^7^, but with several conditions, i.e. stratosphere-troposphere exchange times of 1.5-year and 2.0-year^16^ and ^14^C production share rates between the stratosphere and troposphere (strat:trop = 70%:30%, 80%:20%, and 90%:10%), we examined Δ^14^C response to a square pulsed ^14^C input (single-pulsed event). Three free parameters of pulse duration, pulse height, and, pulse start date for single-pulsed events were estimated by fitting calculation to the Δ^14^C data using the box model. Details of the box model and the fitting calculation are described in the methods section. The fitting results are shown by contour maps of reduced χ_ν_^2^ values in the Supplementary Information (Fig. S3). Figure 3 shows the contour map containing the smallest reduced χ_ν_^2^ value among all the six conditions, i.e., the exchange times of 1.5 years and the share rate (70%:30%) in the stratosphere-troposphere. In this condition, durations from one month to 41 months (3.4 years) cannot be rejected at 95% confidence level, according to the pulse start dates from 665 BCE to 663.5 BCE. The total input ^14^C productions were in a range of (1.3 – 1.5) × 10^8^ [atoms/cm^2^] based on the 95% confidence level. Even with the other conditions, also, the durations were up to 30–39 months in same manner. For any conditions, moreover, the total input ^14^C productions were almost same on the basis of 95% confidence level (Table S1). In the case of the 775 CE event, the best-fitted duration of a square-pulsed ^14^C input is less than a year^1,7^ and longer durations than 1.5 years can be rejected with 95% confidence level^7^; therefore, characteristics of input durations of ^14^C between the 775 CE event and the ~660 BCE event are different, i.e. the ~660 BCE event allows a long-term ^14^C input.

## Discussions

**Figure 4:**
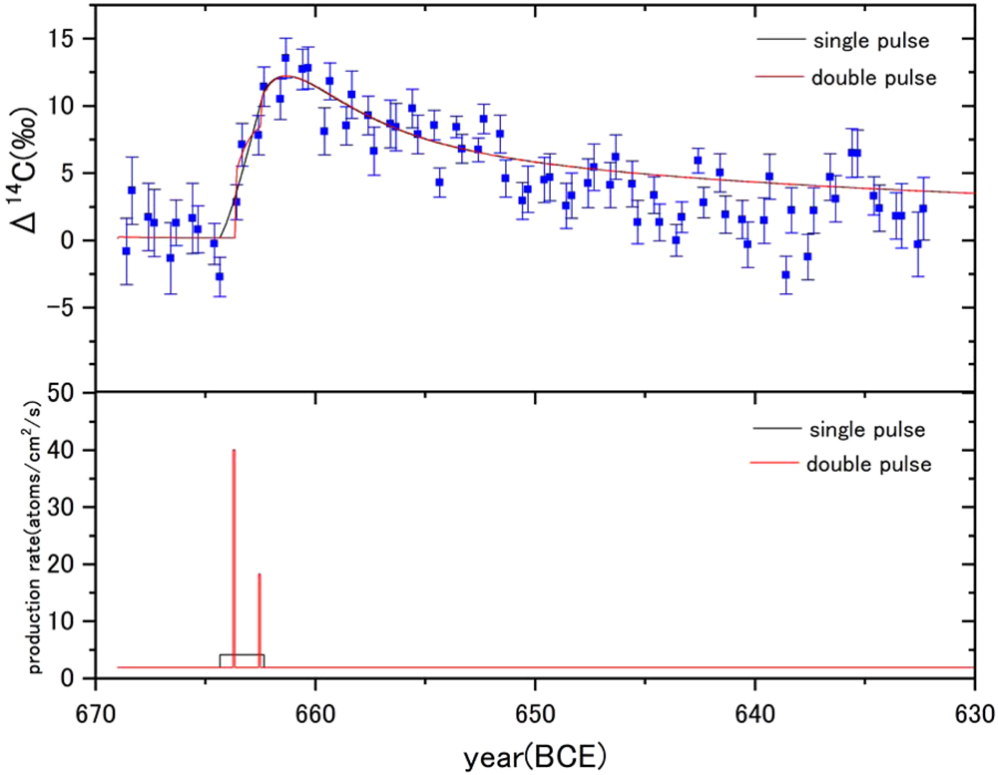
(Upper panel) Best-fitted profile by the 11-box model calculation on the Δ^14^C data set for single-pulsed (black line) and double-pulsed (red line) events. (Lower panel) ^14^C production rates injected in the atmosphere and achieving the best-fit profile of the Δ^14^C in the upper panel (black line: single-pulsed event; red line: double-pulsed event).

A clear distinction between the ~660 BCE and 775 CE events is the rise times of ^14^C concentrations, which are 3 years and 1–2 year, respectively. It is very important to figure out whether the large ^14^C production is triggered by a single SPE or by multiple SPEs, not only in space–earth environment science but also in solar physics. However, there has been presented no evidence of multiple SPEs for the known rapid ^14^C increase events like the 775 CE event, because multi-annual ^14^C inputs (>1.5 years) associated with the event have been clearly rejected^7^. On the contrary, the ~660 BCE event allows duration up to ~41 months (a few years), and a step like increment profile of ^14^C concentrations is appeared in Fig. 4, which might imply a multiple SPEs case.

A sequence of SPEs originating from the similar activity burst is a scenario of the multiple SPEs case. The example is the ones during July-November 1989, October-November 2003, or January 2005, etc.^19^. Also, a long-separated consecutive-event with an interval up to a few years is another possible scenario of the multiple SPEs case. Hence, we tested a double square-pulsed ^14^C input with an interval (double-pulsed event). Figure 4 shows the best-fitted profile on the Δ^14^C data set and the input ^14^C production rates of double-pulsed event (the fitting details are in Fig. S4 and Methods). The best fitted parameters are the pulse start date of 663.7 BCE, the interval of 14 months, and the total ^14^C production of 1.4 × 10^8^ [atoms/cm^2^], under the conditions of the exchange times of 1.5years and the share rate of 70% and 30% in the stratosphere-troposphere. For the single pulsed-event, we, also, plotted the best-fitted profile and the input ^14^C production rates in Fig. 4 compared with the double-pulsed event. The best fitted parameters are the pulse start date of 664.9 BCE, the pulse duration of 24 months, and the total ^14^C production of 1.4 × 10^8^ [atoms/cm^2^], under the conditions of the exchange times of 1.5years and the share rate of 70% and 30% in the stratosphere-troposphere. Thus, the total ^14^C productions are almost same in both the pulsed-events and the start dates of double-pulsed events are confined in the range of the single-pulsed events (Tables S1 and S2), indicating a possibility of multiple SPEs.

The net production of ^14^C was (1.3 – 1.5) × 10^8^ [atoms/cm^2^] for the single-pulsed event with the same fitting condition to the 775 CE event by Güttler *et al*.^7^, i.e. 2.0-year exchange time and 70%:30% share rate. The estimated production of the ~660 BCE event was 32%~40% smaller than that of the 775 CE event, e.g. 2.2 × 10^8^ [atoms/cm^2^]^7^, but ~15% larger than that of the 994 CE event, e.g. (1.22 ± 0.37) × 10^8^ [atoms/cm^2^]^8^. Suppose the ~660 BCE event was similar to the 775 CE and 994 CE events, then the event scale should be between the two latter events. O’Hare *et al*., also, showed the event scales of the ~660 BCE event by fluences based on ^36^Cl/^10^Be ratios, e.g. F_30MeV_ fluence of the ~660 BCE event is 26 ± 27% smaller than that of the 775 CE event^12^, which is consistent with our result of the ^14^C production ratio, taking account of the error. To compare the event scale, we calculated ^14^C production rates for the contemporary SPEs observed in 1956 and 1972^20,21^ using Phits simulator^22^ and calculation method by Kovaltsov *et al*.^23^. (see Methods). The calculation presents that the net ^14^C production of the ~660 BCE event is equivalent to roughly 50 times larger than that of SPE1956 which is the strongest of the known hard spectrum events in the cotemporary observed SPEs. Consequently, the single-pulse scenario (as proposed in O’Hare *et al*.^12^) is consistent with the new ^14^C data, while a longer production is also possible.

In conclusion, we reveal that it is possible that the ~660 BCE event has rather a long-time ^14^C injection up to 3.4 years in the atmosphere by the finely measured ^14^C data set. The ^14^C measurements of earlywoods and latewoods provide us a constraint on the occurrence time of the SPEs that brought the ~660 BCE event. Our research demonstrates that ^14^C analysis using a finely cut tree ring is advantageous in the field of remarkable cosmic event studies. The estimated scale of the ~660 BCE SPE is much larger than historically recorded SPEs. Our finding indicates that such extreme events might have occurred consecutively within a few years.

## Methods

### Dating of choukai–jindai cedar

Calendar-dating of the outer tree ring in contact with the bark was independently estimated as 477.5 ± 12.5 BCE and 460 ± 8 BCE by a wiggle matching from two kinds of ^14^C measurements using liquid scintillation counter and accelerator mass spectrometer (AMS), respectively, indicating the consistent age^24,25^. On the other hand, dendrochronology indicated that a remarkable sector collapse of Choukai volcano occurred at 466 BCE by an analysis of woods dug out of a vicinity of the volcano, using the dendrochronology calibration chart in Japan by Mitsutani^26^. Moreover, debris avalanche deposits related to volcanic eruptions were shown in a volcanology study of Choukai volcano^27,28^. From these, it was inferred that the wood sample was buried by the debris avalanche deposits in 466 BCE in agreement the date of wiggle matching.

### Pretreatments and measurements

Each earlywood and latewood of annual tree rings were separately taken out of a block of boiled wood using tweezers. As the tree-ring was rather thick with typically 3–5 mm width, separating earlywoods and latewoods was not so difficult. Alpha (α)-cellulose was extracted for each separated sample, as it is the most reliable chemical component corresponding to the concentration of ^14^C taken in tree ring cell walls during growth. The α-cellulose yield from the wood sample was approximately 15% by weight. Graphite samples were produced by burning 3 mg of the cellulose. The weight ratios of the graphite to 1 mg of iron powder were kept between 0.6 and 1.2. Measurements of ^14^C in the graphite samples were carried out using a 500 KV tandem accelerator at the Yamagata University (YU–AMS)^29^. The ^14^C concentrations for the graphite samples were obtained using the measured values of the ^14^C/^12^C ratio for samples and 4990 C oxalic acid, which is a standard sample. The measured ratio for a blank sample using IAEA-C1 was typically 1.0 × 10^−15^. Duplicate to sextuple measurements in most of earlywoods and latewoods in 669–633 BCE were performed with the accuracy of approximately 2.3‰ in every single measurement. Reproducibility of the multiplicate measurements was checked by Chi-squared test. Only two out the 70 items in the data set included one rejected ^14^C value at 95% confidence level. Hence, we expressed the measured data set by the weighted averages and the errors in the weighted averages for multiple measurements.

### Difference in oak and cedar physiologies

Oak is a ring-porous angiosperm whose leaves sprout from May and fall down in late October. A row of vessels with a large diameter in oak trees is positioned in an initial portion of earlywoods and its lignification is through within approximately four weeks before leaf-out in the year^30,31^. With this, the photosynthates forming its xylem should be stored for production in the parenchyma cells at the previous year, meaning, the ^14^C concentrations in alpha cellulose constructing the xylem are taken up from the atmosphere of the preceding year. On the contrary, cedar is a type of conifers, which are more primitive plants transporting water through tracheids than angiosperms. In cedars, earlywood is produced in spring and early summer while latewood is formed in late summer^30^. At approximately 350 km south along the Japan sea side from Choukai volcano, the ^14^C contents of a cedar tree ring for 10 years from 1989 were comparable in each year to the observed atmospheric ^14^C concentrations in the area during mid-June and early-September, indicating that the cedar tree ring reflects the ^14^C concentration in the atmosphere of the year it grows^32^.

### Calculation of ^14^C Production rate

Figure S5 depicts an exponential function of the Δ^14^C profile fitted to the ^14^C data set of earlywoods and latewoods using least squares method. The decay constant was 13.0 ± 1.4 years, which is comparable to the value 14.2 years from a contemporary Spanish pine tree ring due to atomic detonations in the 1960s^33^. It evidently indicates a process of carbon cycle in which a pulsed ^14^C is injected into the atmosphere, and then the ^14^C are dissolved in the surface ocean. We simulated the Δ^14^C profile for pulsed ^14^C productions using the 11-box model^7^. The calculation procedures were almost similar to that of Güttler *et al*.^7^, except that we used ^14^C half-life of 5730 years. We used fluxes between boxes in Fig. 3 of Güttler *et al*.^7^, and initial state of ^14^C inventories in Table 1 of Güttler *et al*.^7^. In the simulation, we examined the pulsed ^14^C productions for the two kinds of exchange times of 1.5 years and 2.0 years^16^ between the stratosphere and troposphere, taking account of the three cases of the ^14^C productions shared into two boxes of the stratosphere and troposphere, i.e. stratosphere:troposphere = 70%:30%, 80%:20%, and 90%:10%.

The fitting calculation was conducted for 30 Δ^14^C data of earlywoods and latewoods between 669 BCE and 655 BCE. We assumed the growth periods of earlywoods and latewoods as April–July and August–September, respectively; therefore, the measured Δ^14^C values of earlywoods and latewoods were compared through averaging the simulated values of the two periods. The validity between the measured and simulated data sets was evaluated using reduced χ_ν_^2^ values (χ^2^/ν, ν: degree of freedom). The injected pulse shape was simplified to a square pulse of ^14^C production in the atmosphere. Two types of pulsed inputs were examined, which indicate a single pulsed event and a double pulsed event with an interval. Three fitting parameters coming down to the dof = 27 are employed in the single pulse input, i.e. pulse height, pulse width, and pulse start time, which express total ^14^C production, duration, and beginning time for a single-pulsed event, respectively. In the double pulsed input, four fitting parameters coming down to the dof = 26 are employed, i.e. pulse heights of the first and second pulses, time interval between the first and second pulses, and the first pulse start time. For the double pulsed event, we simply fixed to one month the pulse widths of the first and second pulses. The pulse width, the beginning time of the event, and the time interval were shown with a month resolution. The fitting results are shown in Figs. S3 and S4. Table S1 shows the fitting results for each condition.

### ^14^C Production rate by SPEs

We calculated an altitude distribution of ^14^C production up to 40 km in the atmosphere with the proton energy range of 20 MeV to 5 GeV using Phits simulator^22^. The resulting ^14^C yield function was very consistent with the previous works^23,34^. From the approximately 60 GLE events since 1956, two GLE events of SPE1956 and SPE1972 were chosen as typical SPEs representing hard and soft energy spectra, respectively^20,21^. The net ^14^C productions by SPE56 and SPE 72 are 2.8 × 10^6^ [atoms/cm^2^] and 6.2 × 10^5^ [atoms/cm^2^], respectively, calculated at every latitude taking account of a geomagnetic cut off rigidity^35^ from their fluence spectra and the altitude production yields. The production rate of SPE56 was consistent with the value 2.90 × 10^6^ [atoms/cm^2^] by Kovaltsov *et al*.^23^. Based on our calculation, the ~660 BCE event is 52–53 times larger than SPE56, and 230–240 times larger than SPE72 if the energy spectrum of the ~660 BCE event is comparable to the SPEs.

### Data set

The data set that supports the findings of this study is shown in Table S3.

## Supplementary information

Supplementaryinformation.
