# Supplementary material for: Prolonged production of 14C during the ~660 BCE solar proton event from Japanese tree rings
Source: Sci Rep. 2020 Jan 20;10:660. doi: 10.1038/s41598-019-57273-2 (PMC6971252; doi:10.1038/s41598-019-57273-2)
Supplement: Supplementary file 1 — Supplementaryinformation. [file 41598_2019_57273_MOESM1_ESM.pdf]

Supplementary Information

Prolonged production of ^{14}C during the ~660 BCE solar proton event from Japanese tree rings

Hirohisa Sakurai [1], Fuyuki Tokanai [1], Fusa Miyake [2], Kazuho Horiuchi [3], Kimiaki Masuda [2], Hiroko Miyahara [4], Motonari Ohyama [5], Minoru Sakamoto [6], Takumi Mitsutani [7], Toru Moriya [1]

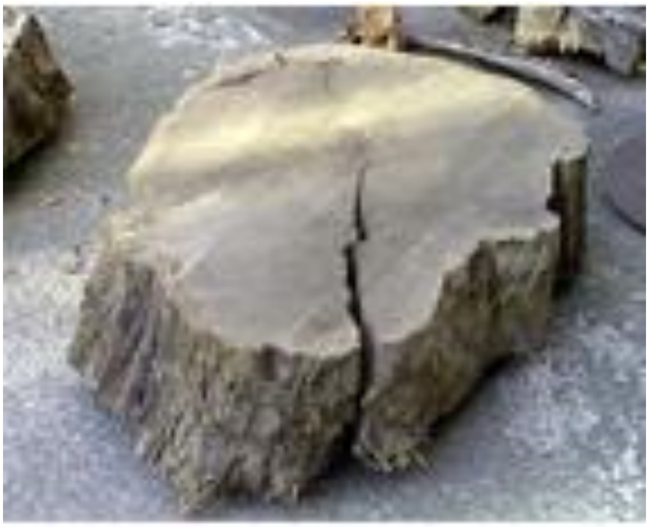

Fig. S1: A photograph of the Choukai-Jindai Cedar (Sakurai et al. 2006, Takahashi et al. 2010). The wood was dug out from a foot of Choukai volcano located at the northern Japan (39°05' N, 140°03' E) in 1996. The Choukai–Jindai Cedar contains approximately 320 tree rings. The wood sample was so well preserved under the ground that the clear barks were retained on the outside. The outermost ring was dated to 466 BCE from a dendrochronological record.

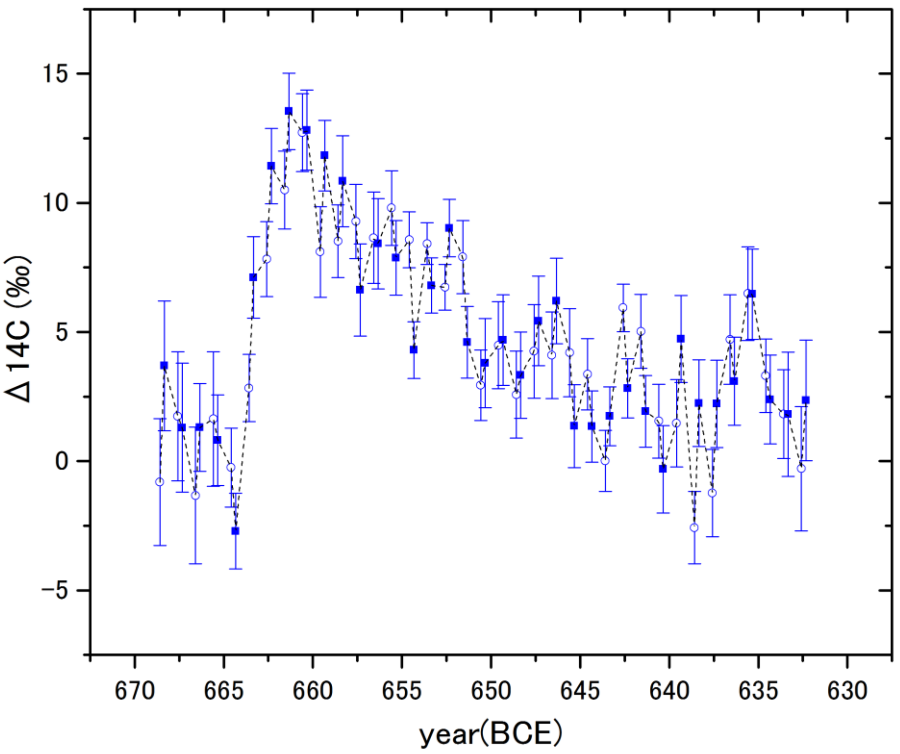

Fig. S2: Measured ^{14}C concentrations in earlywoods (open circles) and latewoods (solid squares) of annual rings of the Choukai–Jindai Cedar for 669–633 BCE.

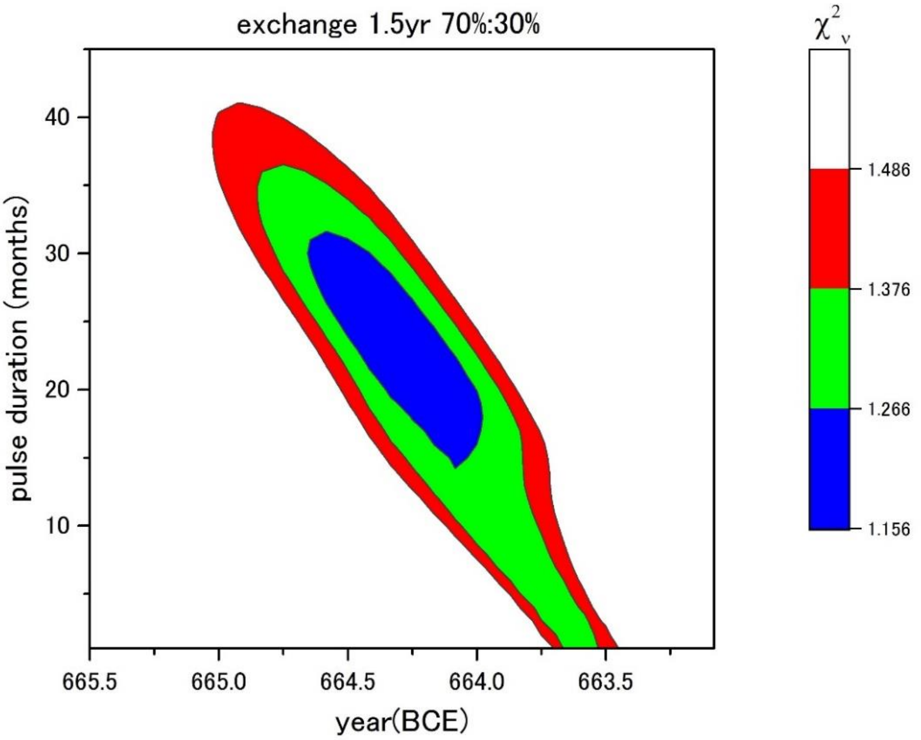
exchange 1.5yr 70%:30%
pulse duration (months)
40
30
20
10
665.5
665.0
664.5
664.0
663.5
year(BCE)
χ^2_ν
1.486
1.376
1.266
1.156

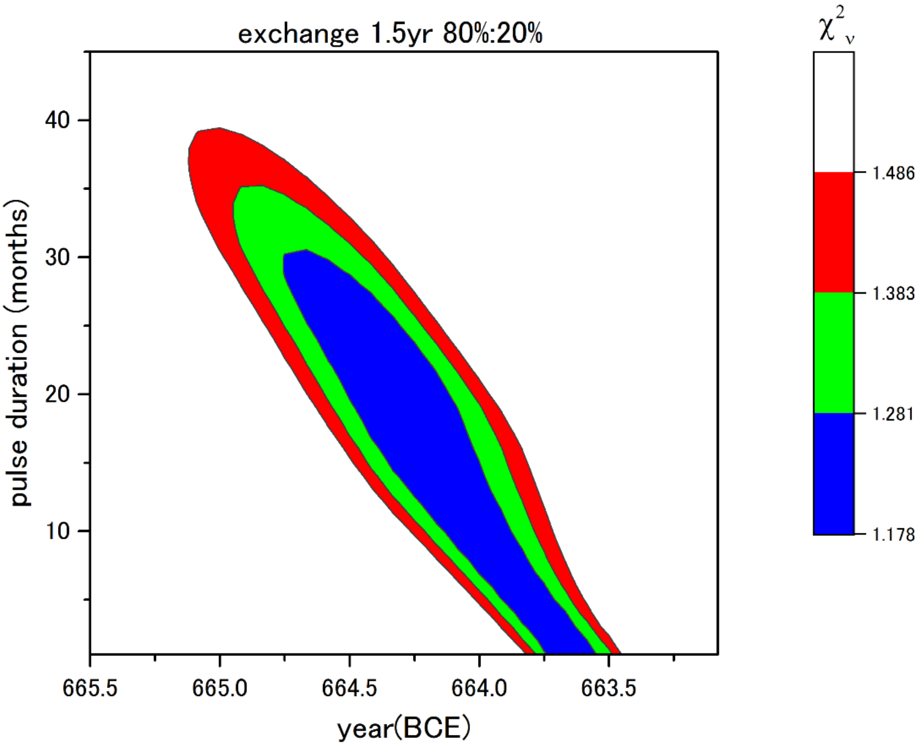
exchange 1.5yr 80%:20%
pulse duration (months)
40
30
20
10
665.5
665.0
664.5
664.0
663.5
year(BCE)
χ^2_ν
1.486
1.383
1.281
1.178

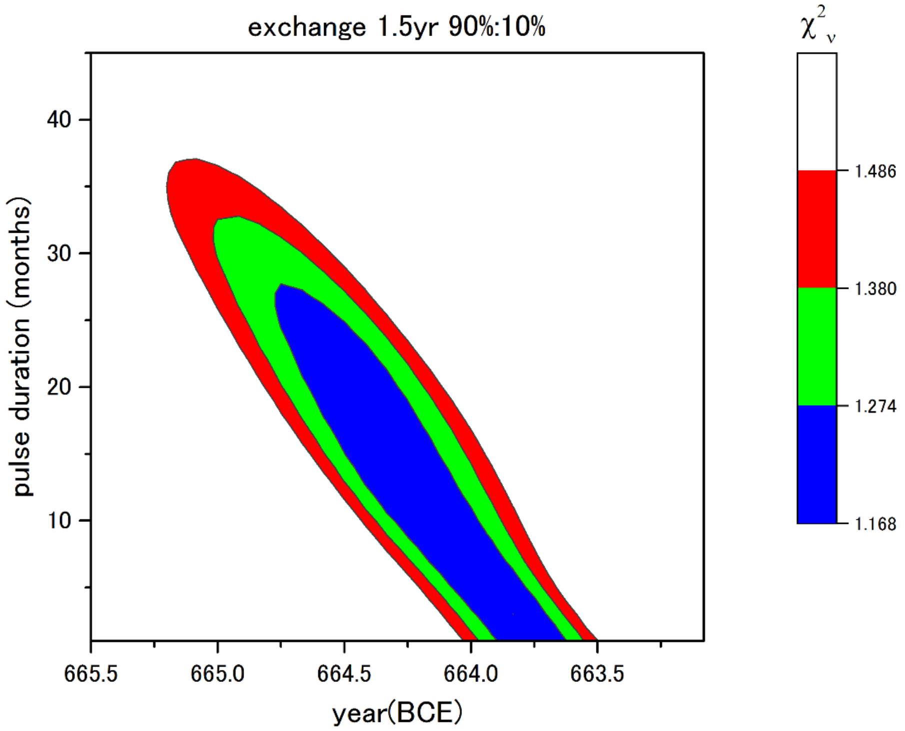
exchange 1.5yr 90%:10%
pulse duration (months)
year(BCE)
40
30
20
10
665.5
665.0
664.5
664.0
663.5
χ^2_ν
1.486
1.380
1.274
1.168

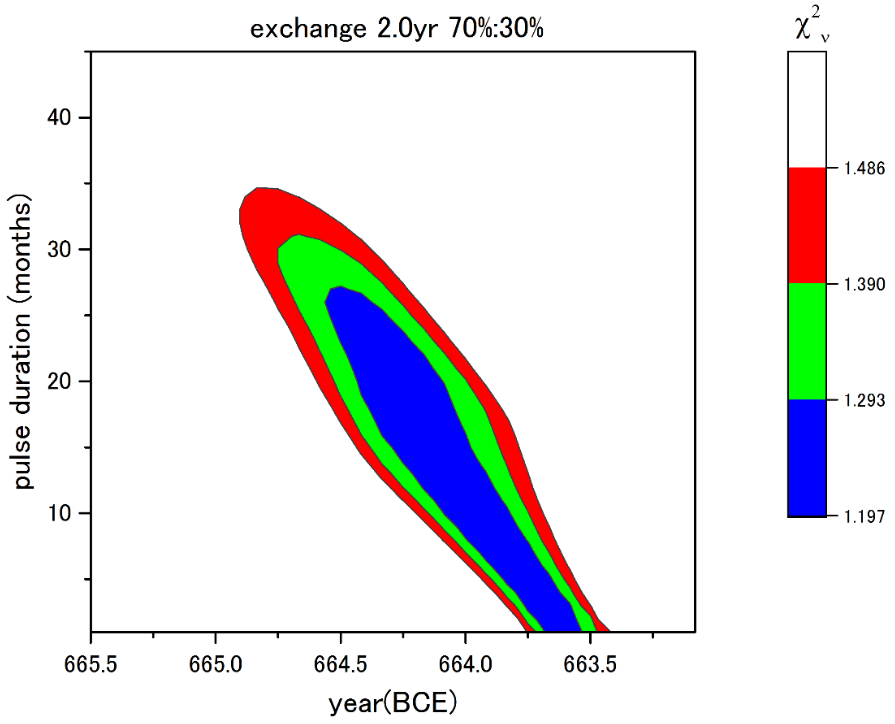
exchange 2.0yr 70%:30%
pulse duration (months)
year(BCE)
40
30
20
10
665.5
665.0
664.5
664.0
663.5
χ^2_ν
1.486
1.390
1.293
1.197

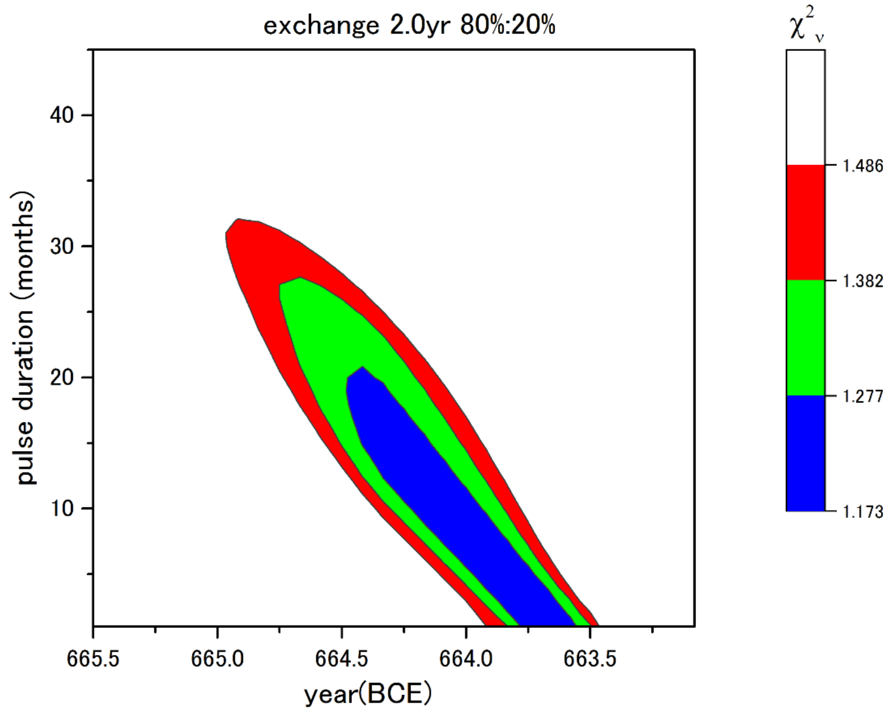

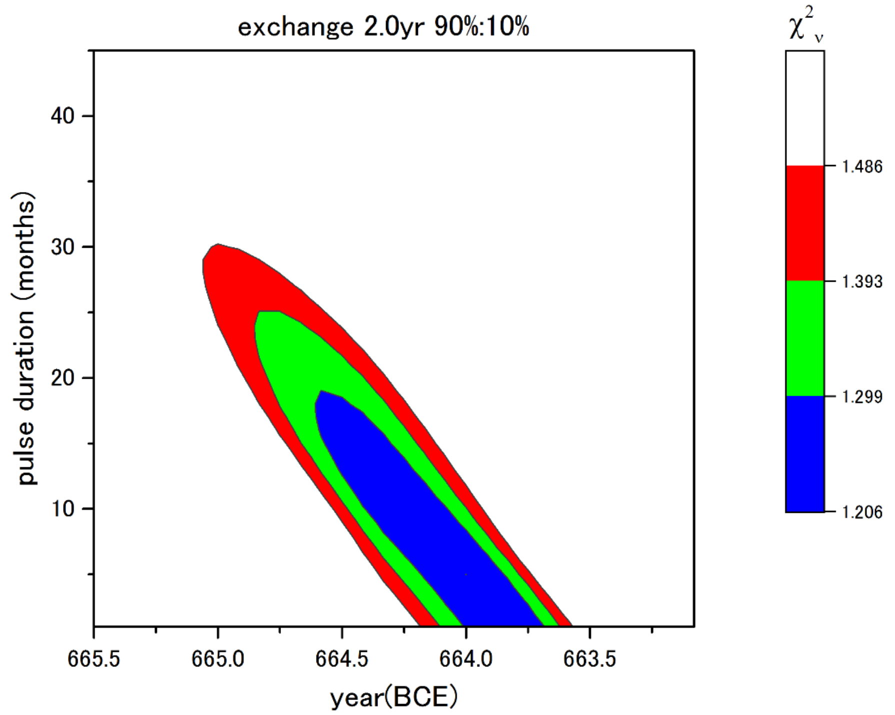

Fig. S3: The fitting results using the 11-box model (single-pulsed event). These contour maps are the best fitted pulse height for each condition, i.e. the exchange time between stratosphere and troposphere: 1.5-year and 2.0-year, and the ^{14}C input production share rate between stratosphere and troposphere: 70%:30%, 80%:20%, and 90%:10%.

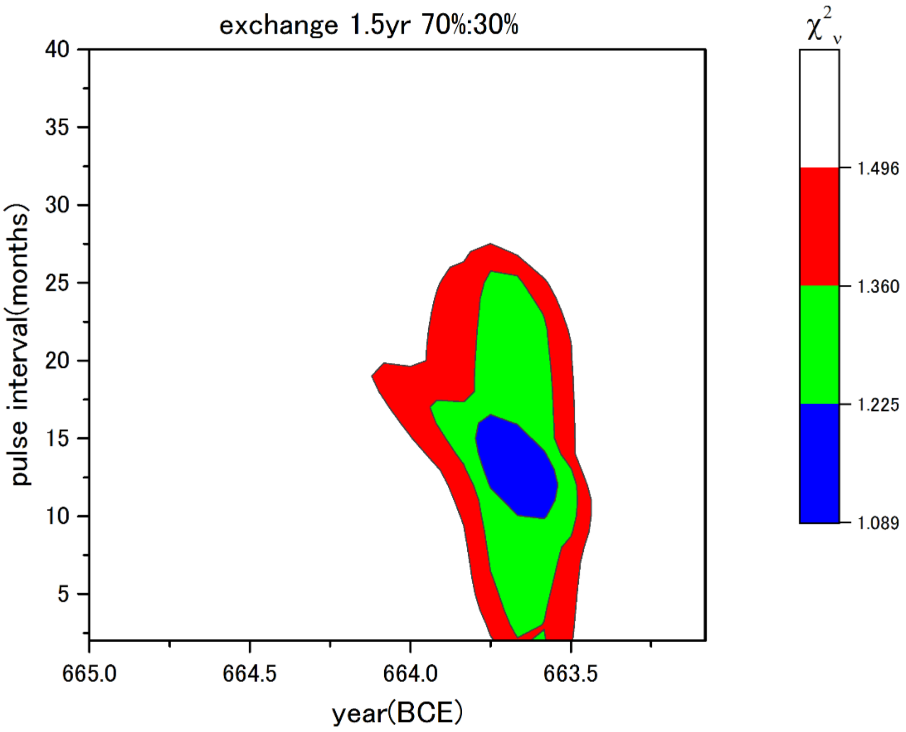
exchange 1.5yr 70%:30%
pulse interval(months)
year(BCE)
40
35
30
25
20
15
10
5
665.0
664.5
664.0
663.5
χ^2_ν
1.496
1.360
1.225
1.089

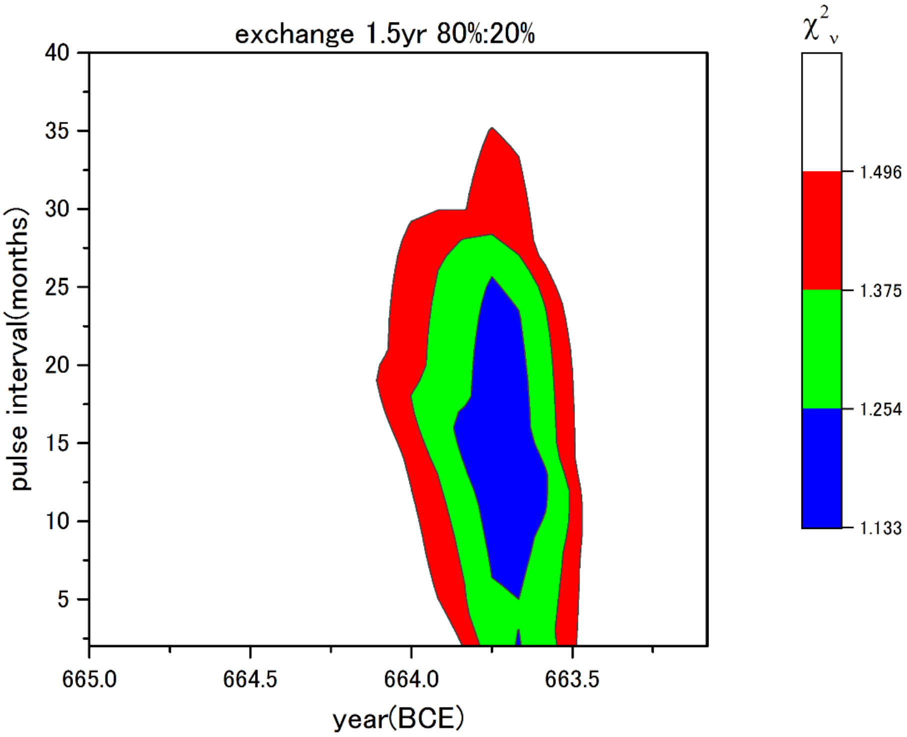
exchange 1.5yr 80%:20%
pulse interval(months)
year(BCE)
40
35
30
25
20
15
10
5
665.0
664.5
664.0
663.5
χ^2_ν
1.496
1.375
1.254
1.133

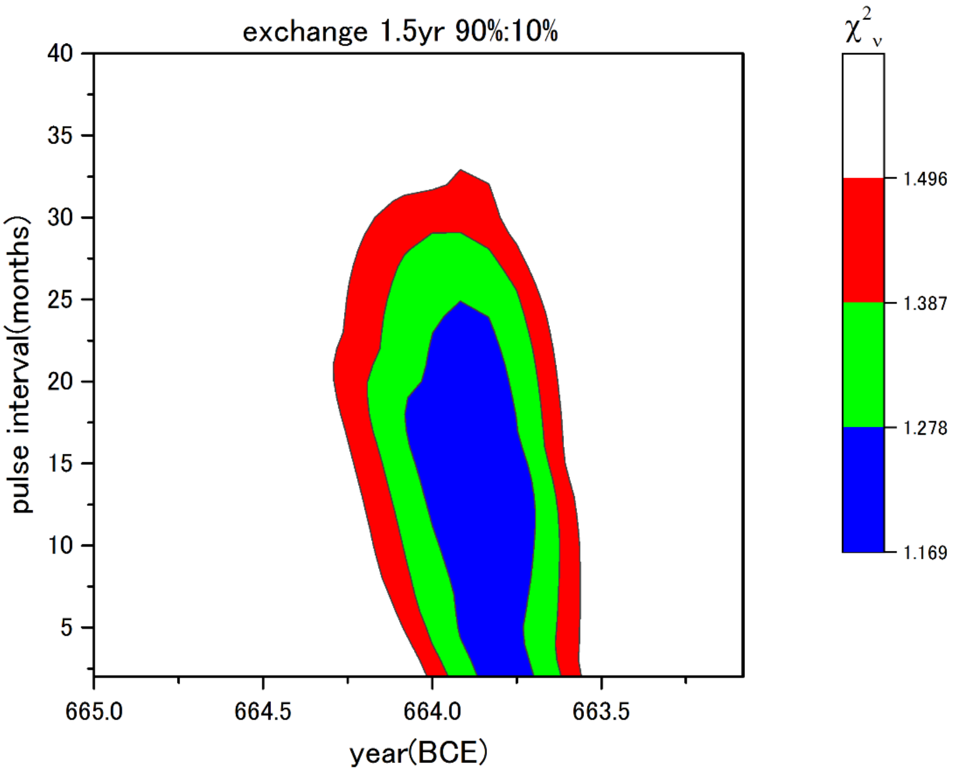

Fig. S4: The fitting results using the 11-box model (double-pulsed event). The X-axis and Y-axis indicate the timing of the first pulse and the length of the interval between two pulses, respectively. These contour maps are the best fitted pulse height for each condition, i.e. the exchange time between stratosphere and troposphere: 1.5-year, and the ^{14}C input production share rate between stratosphere and troposphere: 70%:30%, 80%:20%, and 90%:10%.

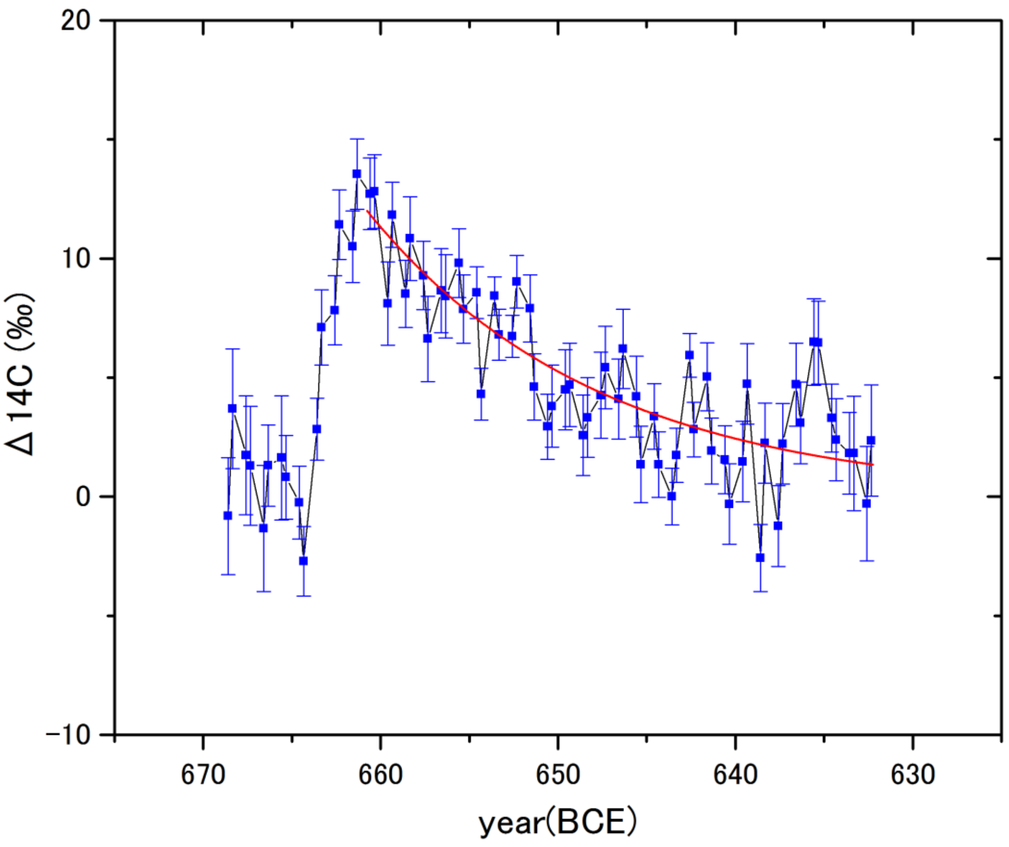

Fig. S5: The red line expresses an exponential function fitted to the dataset of earlywoods and latewoods with a least squares method. It indicates that injected ^{14}C in the atmosphere with a short term are dissolved in the ocean by a carbon cycle process.

Table S1: Obtained fitted parameters for the single-pulsed event. The ranges of parameters show 95% confidence level.

Condition		Event start date [BCE]	Duration [month]	Production rate [10^8 atoms/cm^2]
Exchange rate [year]	Share rate [t:s]			
1.5	70:30	663.5-665.0	1-41	1.3-1.5
1.5	80:20	663.5-665.1	1-39	1.3-1.5
1.5	90:10	663.5-665.2	1-37	1.3-1.5
2.0	70:30	663.5-664.8	1-34	1.3-1.6
2.0	80:20	663.5-664.9	1-32	1.3-1.6
2.0	90:10	663.6-665.0	1-30	1.3-1.6

Table S2: Obtained fitted parameters for the double-pulsed event. The ranges of parameters show 95% confidence level. Durations of first and second pulses are fixed to 1 month.

Condition		Event start date of first pulse [BCE]	First pulse	Second pulse	Pulse interval [month]
Exchange rate [yr]	Share rate [t:s]		Production rate [10^8 atoms/cm^2]	Production rate [10^8 atoms/cm^2]	
1.5	70:30	663.5-664.1	0.99-1.1	0.25-0.47	2-27
1.5	80:20	663.5-664.1	1.1-1.1	0.13-0.45	2-35
1.5	90:10	663.6-664.3	1.1-1.2	0.13-0.30	2-32

Table S3: Measured data set of ^{14}C in earlywoods and latewoods of annual rings of the Choukai–Jindai Cedar for 669–633 BCE. See the Methods for Δ^{14}C and error.

Year (BCE)	Earlywood/Latewood	Δ^{14}C(‰)	error(‰)
669	Ew	-0.8	2.5
669	Lw	3.7	2.5
668	Ew	1.7	2.5
668	Lw	1.3	2.5
667	Ew	-1.3	2.6
667	Lw	1.3	1.7
666	Ew	1.6	2.6
666	Lw	0.8	1.8
665	Ew	-0.2	1.5
665	Lw	-2.7	1.5
664	Ew	2.8	1.3
664	Lw	7.1	1.6
663	Ew	7.8	1.5
663	Lw	11.4	1.5
662	Ew	10.5	1.5
662	Lw	13.5	1.5
661	Ew	12.7	1.5
661	Lw	12.8	1.6
660	Ew	8.1	1.8
660	Lw	11.8	1.4
659	Ew	8.5	1.4
659	Lw	10.8	1.8

658	Ew	9.3	1.4
658	Lw	6.6	1.8
657	Ew	8.7	1.8
657	Lw	8.4	1.8
656	Ew	9.8	1.4
656	Lw	7.9	1.4
655	Ew	8.6	1.1
655	Lw	4.3	1.1
654	Ew	8.4	0.8
654	Lw	6.8	1.1
653	Ew	6.7	0.9
653	Lw	9.0	1.1
652	Ew	7.9	1.4
652	Lw	4.6	1.4
651	Ew	2.9	1.4
651	Lw	3.8	1.7
650	Ew	4.5	1.7
650	Lw	4.7	1.8
649	Ew	2.6	1.7
649	Lw	3.3	1.7
648	Ew	4.3	1.8
648	Lw	5.4	1.7
647	Ew	4.1	1.7
647	Lw	6.2	1.7
646	Ew	4.2	1.7
646	Lw	1.4	1.6
645	Ew	3.4	1.4
645	Lw	1.3	1.4
644	Ew	0.0	1.2
644	Lw	1.7	1.1
643	Ew	5.9	0.9
643	Lw	2.8	1.1
642	Ew	5.0	1.4
642	Lw	1.9	1.4
641	Ew	1.5	1.4
641	Lw	-0.3	1.7
640	Ew	1.5	1.7

640	Lw	4.7	1.7
639	Ew	-2.6	1.4
639	Lw	2.2	1.7
638	Ew	-1.2	1.7
638	Lw	2.2	1.7
637	Ew	4.7	1.7
637	Lw	3.1	1.7
636	Ew	6.5	1.8
636	Lw	6.5	1.7
635	Ew	3.3	1.4
635	Lw	2.4	1.7
634	Ew	1.8	1.7
634	Lw	1.8	2.4
633	Ew	-0.3	2.4
633	Lw	2.4	2.3
